# The Treatment of Psoriasis

**Published:** 1908-12-05

**Authors:** 


### Dermatology.

### THE TREATMENT OF PSORIASIS.

There is no doubt that internal medication may sometimes exercise a powerful curative effect on psoriasis. But the results obtained by the use of drugs given internally are always uncertain, and less rapid than those obtained by external medication alone.

Internal medicaments operate either by their influence on the nutrition of the skin or by inducing alterations in the general nutrition. Their curative action depends upon the intensity of the physiological changes which they lead to, and it is always necessary to push such remedies to the extreme point of tolerance. For this reason they are better avoided, and only out of respect for tradition is it necessary to study some of these internal methods. The drugs which have been most commonly employed are arsenic, iodide of potassium, thyroid extract, and salicin or salicylates.

*Arsenic.*—In the employment of arsenic in psoriasis the drug must be pushed to its full extent of tolerance, and its curative action is not felt until it has been administered for several weeks. Although in some cases it may cause the eruption to disappear, its action is always uncertain, and frequently without any good effect. Its use does not prevent recurrences. The most convenient method of administration is as Fowler's solution. Beginning with three minims (in water) three times a day after meals, the dose is increased by one-minim gradations until a dose of ten minims is reached, always provided the patient exhibits no gastro-intestinal symptoms. Having reached this dose it is again dropped gradually to three minims, and again advanced to ten, as before, and so on. No patient should be put on a long course of arsenic without being carefully watched.

*Iodide of potassium* has been strongly recommended for psoriasis. But its action is very uncertain, and it must be given in very large doses.

*Thyroid extract* has had also many advocates. It undoubtedly cures many cases, but it also is extremely uncertain. The drawbacks to its use are well known, and it should not be given unless the patient can be kept under observation in bed.

*Salicin or salicylates* have been advocated especially in early or spreading eruptions. The dose must be a large one—from 15 to 40 grains, three times a day. Its use has no serious inconveniences, and it is sometimes worth trying in cases where one especially desires to avoid ointments, although the choice may be given to the patient between the less irksome but uncertain internal remedy and the more disagreeable but certain external method of treatment.

There is no case of psoriasis which cannot be cured by external remedies properly and perseveringly applied. Tar is a remedy which has for long been employed and which is still largely used. It may be prescribed in various forms and combinations. The following are simple and useful prescriptions:—

| | | |
|---|---|---|
| ℞ | Ol. Cadini ... ... ... ... | ʒj.—℥ss. |
| | Vaselin. ad ... ... ... ... | ℥j. |
| ℞ | Picis Carbonis præparatæ ... ... | ʒj.—℥ss. |
| | Vaselin. ad ... ... ... ... | ℥j. |
| ℞ | Ol. Cadini vel Picis Carb. præp. ... | ʒij.—℥ss. |
| | Saponem mollem ad ... ... | ℥j. |
| ℞ | Liq. Picis Carbonis, B.P. | |

Before the use of an ointment the scales should be removed so far as possible by soaping and by soaking in a warm bath. The tar and soap mixture is best employed just before the bath. Both ointments and soap mixture must be well rubbed in. The alcoholic solution of coal tar is painted on to limited areas. When tar applications are being used over large areas the urine should be watched, and if any darkening occurs (indicating toxæmia, which is rare) the application must be stopped.

Although with many tar is the favourite remedy for psoriasis, another drug—chrysarobin—which is also commonly employed, is much more efficacious and more rapid in its action. Its use is attended by certain drawbacks. It stains the linen, and the stains do not wash out. If it gets into the eyes it causes a violent and painful conjunctivitis, and it is also liable to produce acute erythema at the thinner parts of the skin, such as at the flexures of the joints. These evil effects can be avoided by clothing the patient in old garments, by forbidding the application of the remedy to the scalp, face, and hands, and, except with care, to the flexures. The simplest way of applying chrysarobin is in an ointment with vaselin. We may begin with gr. x. to the ounce, and increase to ʒj to the ounce for obstinate patches. The effect of the chrysarobin application is to stain the skin around the patches, while the patches themselves become less scaly and less red, and finally appear as pale areas in the stained and reddened skin around. When they reach this condition the application may be discontinued. For the scalp and hands ointments containing tar, or the following—napthol, 20 grains; resorcin, 20 grains; vaselin, ℥j, which is more cleanly and odourless—may be used. For the scalp a preliminary shampoo, using Hebra's spiritus saponis alkalinus as a soap, removes the scales and favours the application of ointment.

